# A Full-Blown Case of Bronchiectasis: Kartagener Syndrome Without Infertility Diagnosed Later in Life

**DOI:** 10.7759/cureus.1678

**Published:** 2017-09-11

**Authors:** Madeeha Subhan, Waleed Sadiq

**Affiliations:** 1 Capital Hospital Islamabad, Ayub Teaching Hospital, Abbottabad; 2 Department of Medicine, Shifa International Hospital

**Keywords:** kartagener syndrome, infertility, bronchiectasis

## Abstract

Kartagener syndrome (KS) is a rare autosomal recessive genetic ciliary disorder characterized by situs inversus, chronic sinusitis, bronchiectasis, and infertility. KS is associated with ultrastructural anomalies of the cilia in epithelial cells covering the upper and lower respiratory tracts and spermatozoa flagella. This case describes a patient with KS with situs inversus and sudden onset bronchiectasis with a sharp decline in respiratory function presenting later in life but without sinusitis or infertility.

## Introduction

Kartagener syndrome (KS), also known as immotile cilia syndrome, is an autosomal recessive condition that results from a defect in the dynein arm. The immotile cilia seen in KS is due to defects in the ultrastructural organization of the cilia [1-3]. KS presents with the classic symptoms of situs inversus, bronchiectasis, and polyposis nasi.

## Case presentation

A 59-year-old man presented to the emergency department after a road traffic injury in which he was a restrained driver. Trauma protocol was applied, and he was investigated for any injuries or bleeding. He had mild bruises on his chest, but there were no other notable findings from the physical examination. A chest x-ray revealed situs inversus with no rib fractures. Due to this incidental finding, he was asked about any other systemic symptoms. The patient then reported having a fever and cough for the past two months for which he took over the counter medications (which did not help those symptoms). The fever was low grade, gradual in onset, remitting in nature with peaks in the evening, and was occasionally associated with rigors and chills. He also had a cough that was productive with whitish sputum. Sputum production gradually increased from less than half a teaspoon to about two teaspoons. After review, we found no systemic concerns. The patient was troubled since he leads an active life with no comorbidities. The patient is married, has three children, does not smoke, and is not an alcoholic. His family history was positive for diabetes mellitus. On examination, his central nervous system was intact. Heart sounds were normal with the point of maximal impulse auscultated on the right side of the chest. On respiratory exam, he had bilateral expiratory rhonchi and bilateral lower lobe inspiratory crepitus. The patient’s bowel sounds were positive, and we noted right hypochondriac fullness with no visceromegaly. His blood pressure was 120/80 mmHg, pulse was 89 beats per minute, and his respiratory rate was 20 breaths per minute. On pulmonary function tests, his forced expiratory volume after one second was 50%. During hospital admission, he was given moxifloxacin, and he subsequently improved.

The patient’s complete blood picture and metabolic profile are shown in Tables 1-2.

**Table 1:** Complete blood picture

Hemoglobin	6.21 mmol/L
White blood cells	25.3 x 109/L
Red blood cells	5.6 x 1012/L
Platelets	127.7 x 109/L
Mean corpuscular volume.	84.4 fL

**Table 2:** Complete metabolic profile

Sodium	136 mmol/L
Potassium	4.4 mmol/L
Chloride	103 mmol/L
Urea nitrogen	1.9 mmol/L
Creatinine	79.56 µmol/L
Glucose	6.99 mmol/L
Alanine aminotransferase	0.433 µkat/L
Alkaline phosphatase	6.78 µkat/L
Total bilirubin	10.26 µmol/L

**Figure 1:**
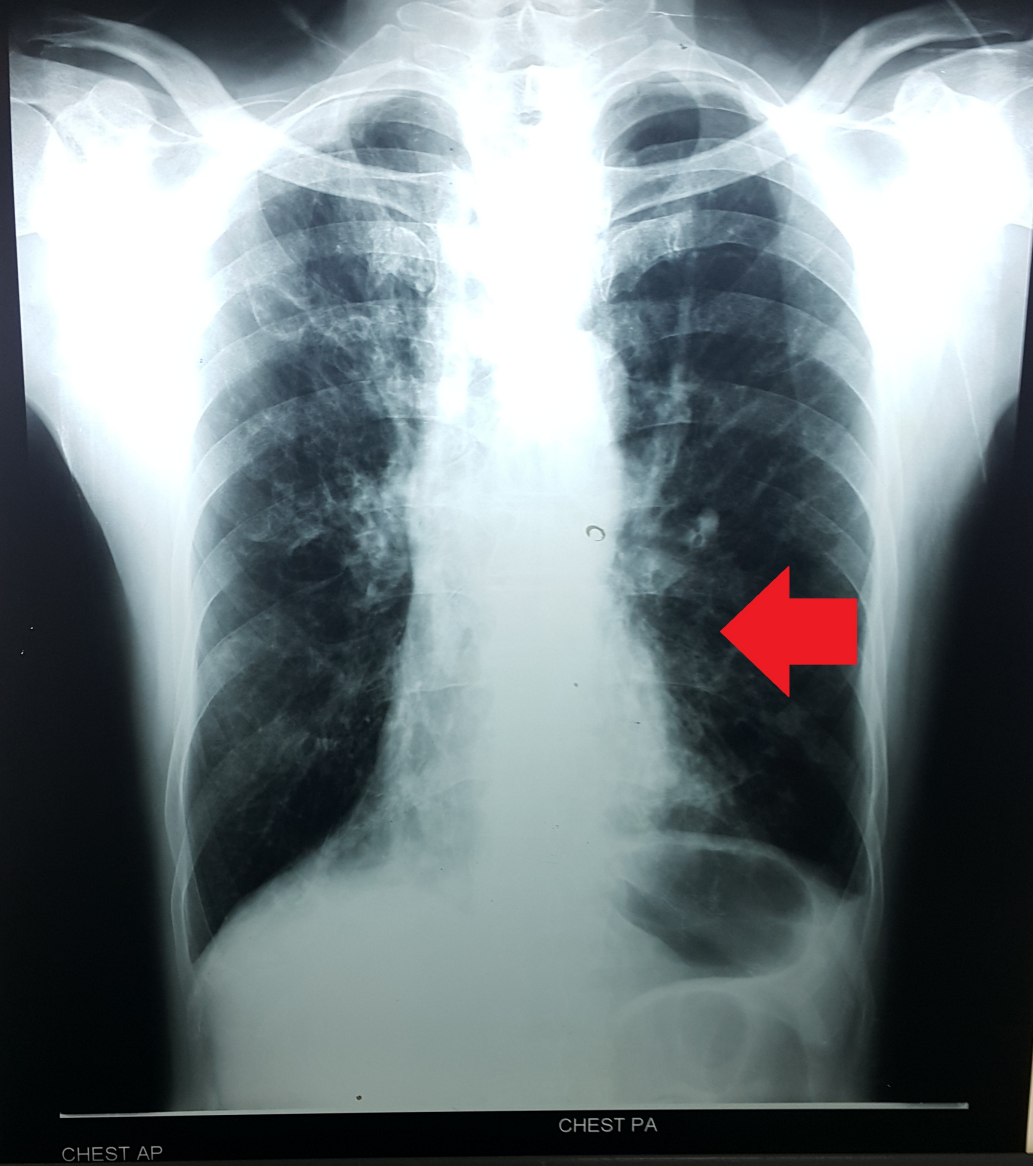
Chest x-ray showing mild cardiomegaly with pulmonary congestion and dextrocardia

On evaluating the patient’s chest x-ray, we noted mild fibrotic changes in both lung fields. We also noted air trapping and flattening of both hemidiaphragms. We also noted increased bronchovascular marking. Cardiac shadow was slightly enlarged with a cardiothoracic ratio of 14/26 cm. The patient had mild cardiomegaly with pulmonary congestion as shown in Figure 1.

The patient underwent a high-resolution computed tomography (CT) scan. We noted mild bilateral apical scarring, bronchiectasis involving both lungs primarily in the right lower and left upper lobes, a mirror image configuration with abdominal situs inversus and dextrocardia. The patient had multiple subcentimeter to borderline benign-looking calcified lymph nodes in the paratracheal, subcarinal, precarinal, preaortic, and bilateral aortopulmonary locations. We noted mild atherosclerotic calcification in the coronary arteries and diffuse generalized osteopenia due to senile osteoporosis.

**Figure 2:**
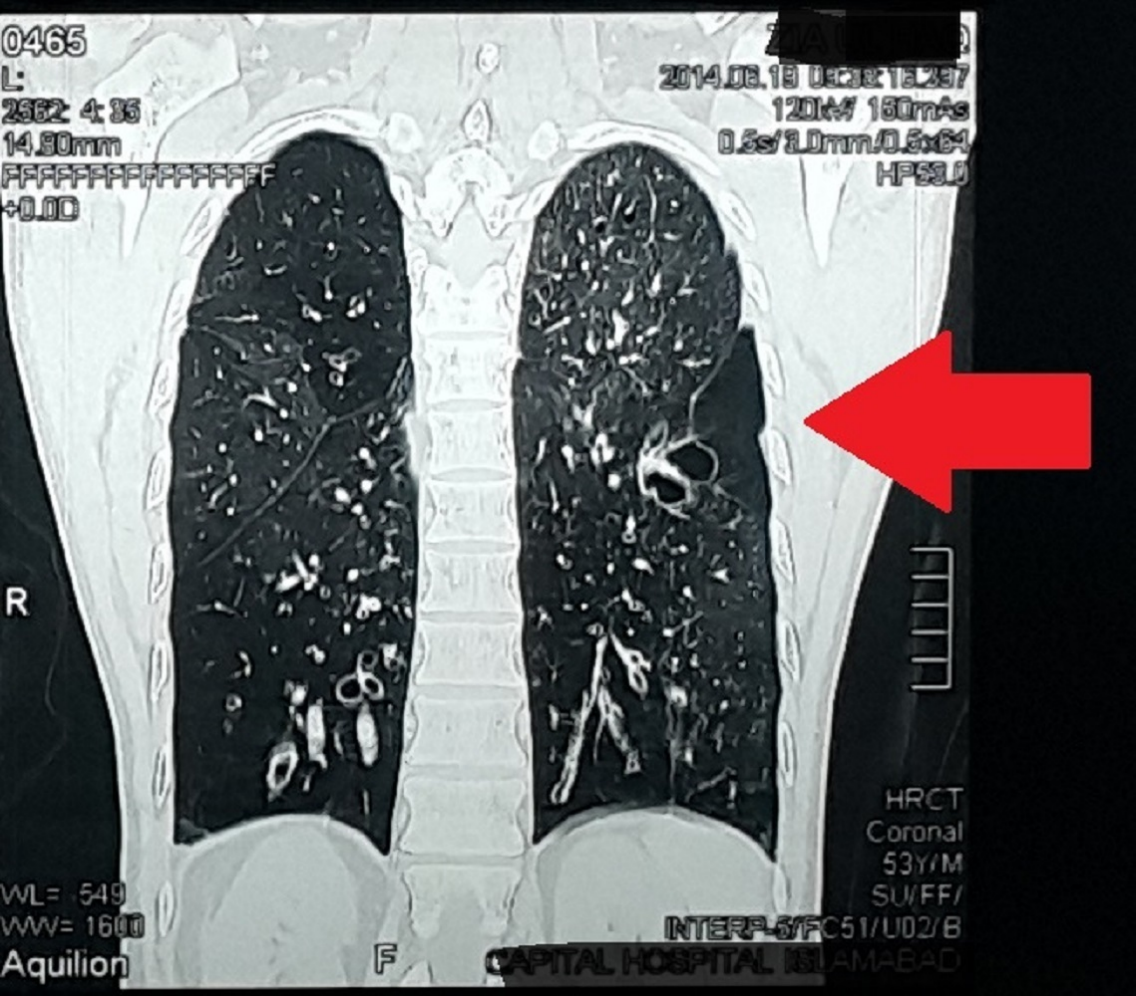
Computed tomography scan showing bronchiectasis

We diagnosed the patient with KS with active bronchiectasis as shown in Figure 2.

## Discussion

KS is a part of primary ciliary dyskinesia occurring in approximately one in 30,000 people [4], but it may range from one in 12,500 to one in 50,000 [5]. Diagnostic criteria are recurrent chest infections, bronchitis, rhinitis since childhood, along with any of the following: situs inversus in the patient or a sibling, alive but immotile spermatozoa, reduced or absent transbronchial mucociliary clearance, and cilia showing characteristic ultrastructural defects [6]. The severity of symptoms is quite variable, although the symptoms are present from birth [7-8]. Our patient presented at age 59, and he was previously healthy with normal fertility and three children. Male patients with KS invariably present with infertility while women have reduced fertility [9]. Infertility in male KS patients is due to diminished sperm motility, and in females, it is due to defective ovum transport [10]. Given our patient’s age and fertility, it is possible that those with KS can have normal fertility and be diagnosed later in life.

## Conclusions

The benefits of early KS diagnosis have yet to be confirmed, but it is likely that an early diagnosis will aid in preserving pulmonary function, quality of life, and life expectancy. However, large prospective studies are needed to validate this theory. KS patients frequently have repeated sinopulmonary infection episodes, for which they have to seek medical attention; this is largely the reason for their morbidity. However, infertility is also an important aspect that needs to be addressed so that they may be offered a suitable option that could help them have children if desired. Moreover, it is important to note that KS patients may present later in life with full-blown bronchiectasis and a sharp decline in pulmonary functions as illustrated in this case.
